# Development and Usability Testing of an Emergency Alert Device for Elderly People and People with Disabilities

**DOI:** 10.1155/2020/5102849

**Published:** 2020-02-20

**Authors:** Suchitporn Lersilp, Supawadee Putthinoi, Peerasak Lerttrakarnnon, Patima Silsupadol

**Affiliations:** ^1^Department of Occupational Therapy, Faculty of Associated Medical Sciences, Chiang Mai University, Chiang Mai, Thailand; ^2^Department of Family Medicine, Faculty of Medicine, Chiang Mai University, Chiang Mai, Thailand; ^3^Department of Physical Therapy, Faculty of Associated Medical Sciences, Chiang Mai University, Chiang Mai, Thailand

## Abstract

The objectives of this study were to develop and evaluate the effectiveness of an emergency alert device for elderly people and people with disabilities by usability testing. There were two phases in this study: (1) development of a prototype for an emergency alert device and (2) usability testing of the device. Results presented development of the prototype, which comprised parts for sending and receiving signals. There were two kinds of alarms for emergency calls known as conscious and unconscious alerts. Participants in the usability testing phase included 12 specialists and 161 users that comprised 146 elderly people or people with disabilities and 15 caregivers or community health volunteers. The instruments used were a rating scale, usability checklist, and individual interviews regarding the usability, general appearance, and use of the device. The users agreed with the overall aspects regarding usability of the device, its general appearance, and use ($\bar{\text{X}}$ ± **SD** = 4.24 ± 0.88, 4.11 ± 0.90, and 4.37 ± 0.83, respectively). Most of the participants, both specialists and users, gave their perspectives on improving the size, color of the letters displayed, type of wristband, and method for sending signals.

## 1. Introduction

The number of dependent people in Thailand is increasing, particularly among elderly people and people with disabilities [1, 2]. In 2017, the Foundation of Thai Gerontology Research and Development Institute (TGRI) reported that Thailand will be an aging society by 2022 and a completely aged society by 2037 [2]. The impact of this situation will create an increasing number of people with disabilities because elderly people are at risk of falling down frequently, having accidents at home, and contracting diseases more than other age groups. In the past, Thai people lived in extended families, in which the family cared for their elderly or dependent members. Now, there is a growing number of nuclear families in the working age group that tend to move to the city. Dependent people, including the elderly and people with disabilities, are finding a lack of caregivers in the community, and so some members of the working age group have decided to give up their jobs to care of the dependent people in their family [3]. Unfortunately, this situation brings about loss of income, no time for leisure or social participation, low self-esteem, and high stress levels.

Assistive technology (AT) is a way of enhancing the quality of life of dependent people and their caregivers [4–6]. AT is developed and designed functions to increase performances of elderly people and people with disabilities in activities of daily living, work, leisure, education, and social participation. Therefore, with the increasing number of dependent people and lack of caregivers in the community, application of AT is necessary in helping the elderly live safely at home and in their community, and it also helps caregivers to have balanced daily activities. Previous studies by the authors [3, 7, 8] found that most of the elderly people and people with disabilities in the community worried about living alone safely, but did not want family members to give up their job to be their caregivers. However, the working group informed that they felt happy to take care of their family members, most of which were their parents, but sometimes they wanted to have free time to do part-time jobs and participate in social activities without the worry of their dependents living alone.

Therefore, various types of emergency alert devices have been developed recently for dependent people, including children, people with disabilities, sick patients, and the elderly. The purpose of these devices is to locate the persons involved and monitor their safety and security and increase their quality of life [9–11]. Most of them send out an alarm only when help is requested, but some are developed to monitor the health of sick patients such as their blood pressure, heart rate, and so on. These health records and daily activity data are collected usually in digital format and analyzed for detecting the risk of serious health problems and sharing between health professionals [9, 11]. In addition, current technologies are able to use sensors, real-time cameras, and smart phones as supplementary alert devices for a surveillance system [9, 12, 13].

Therefore, this study aimed to develop an emergency alert device for the safety of elderly people and people with disabilities while their caregivers leave them alone. Although many alert devices are available in stores, most of them are set up and used in hospitals, or permanently positioned in the home, and cannot be carried with the user. While some devices are movable, most of them usually monitor only falls or movement. As a result, this study also aimed to develop a low-cost prototype device that can give alarm when elderly people and people with disabilities need help or have no movement for periods of time, possibly due to unconsciousness. This choice of device is for dependent people who live alone at home and their caregivers who need to perform outside activities for a duration. With this device, dependent people and their caregivers can contact each other at times of emergency. Therefore, elderly people and people with disabilities as well as their caregivers will have increased quality of life in the community.

## 2. Methods

This study had a research and development design approved by the Research Ethics Committee of the Faculty of Associated Medical Sciences, Chiang Mai University, Thailand (AMSEC-61EX-001). It comprises two phases: (1) development of a prototype for the development of an emergency alert device and (2) usability testing of the device for elderly people and people with disabilities in the community. The device was designed in the first phase from the literature review and analysis of similar devices recently put on the Thai market. The points of analysis on the device were general appearance, functions, and possible usability by the elderly and disabled people in the community. Then, a prototype of the device was developed by working with the medical engineering team. The second phase used the prototype of the device for consideration by the participants, which included 12 specialists and 161 users. The specialists worked in areas of medical and community services and assistive devices. They consisted of eight occupational therapists, two nurses, a physician, and a physical therapist. The users included caregivers or community health volunteers and elderly people who lived with and without disabilities in Sannameng subdistrict, Chiang Mai, Thailand. Fifteen caregivers or community health volunteers and 146 elderly people, who were interested in the alert device, were recruited by purposive sampling, with the elderly people selected from the elderly school at Sannameng. There were 39 male (24.22%) and 122 female (75.78%) participants. The mean age and standard deviation of the users in this study were 69.42 ± 6.20 years (min. = 60, max. = 91). After the trial period, individual participants were interviewed in-depth, with their answers put on the rating scale usability checklist that comprised two parts: general appearance (six items) and usability aspects (six items). Data were analyzed by descriptive statistics and content analysis.

## 3. Results

### 3.1. Development of a Prototype for an Emergency Alert Device for Elderly People and People with Disabilities

The points of analysis from the literature review and analysis of recent alert devices on the Thai market [7–9] were general appearance of the device, functions, and usability by elderly people and people with disabilities in the community. In terms of general appearance, this study found that most alert devices had at least two parts: a signal sending part and signal receiving part. Some sending parts were designed with a wristband or like a wristwatch or message pager, but others were designed as a box that was fixed on the wall. However, all of the designs had an SOS button. Most of the receiving parts were designed as an alarm box, with a sound alarm, but some had sound and real-time video calls. In terms of functions, the alert devices could be categorized into two groups: emergency alertness and movement detection. Most of them had an SOS or a panic button, by which the elderly or disabled users were able to request help when they felt uncomfortable or unsafe. Some emergency signals sent sound and pictures through the Internet cloud server to smart phones of relations, but others went to the nurse station. This function was used by persons who were conscious. In addition, some devices worked as a movement detector by using a sensor monitoring system. This was a way of helping dependent people in cases of falling and inability to move or press the SOS button. Moreover, the function of movement detection in some devices included space limitation for elderly people in the home, particularly those with dementia or Alzheimer's disease, who might stray outside the house. However, movement detection was set up usually in hospitals or institutes so that emergency signals could be sent to a central monitor in the nurse station or to the nurses' pager. Regarding the possible usability by elderly and disabled people in the community, most devices were designed with easy-to-use and noncomplex steps. However, expansion of these types of devices did not occur. As a result, the prototype of the emergency alert device in this study was designed and developed as follows.

#### 3.1.1. General Appearances of the Device

**Figure 1:**
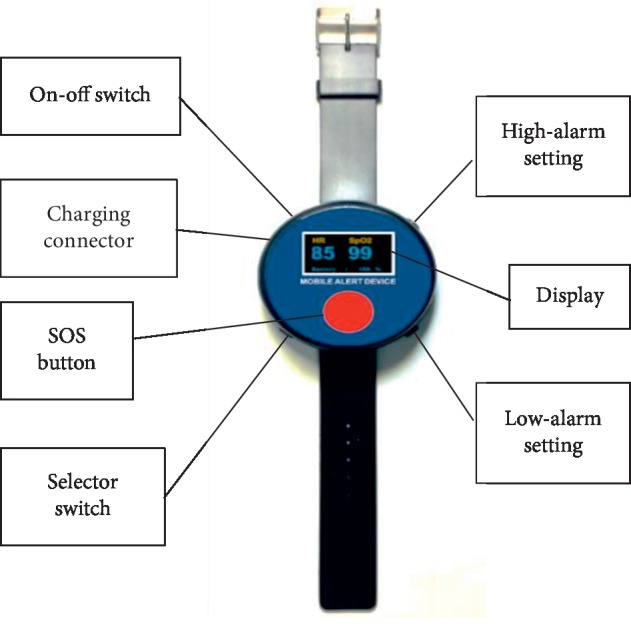
The signal sending part of the emergency alert device.

The prototype of the emergency alert device comprises two parts: a signal sending part and signal receiving part. The signal sending part looked like a wristwatch with a wristband and a large red SOS button on the dial (as shown in Figure 1). There were seven subparts in this part such as an On-Off switch, charging connector for battery charging, display, SOS button, selector switch, and high-alarm and low-alarm setting. The display showed the active status, percentage of remaining battery strength, heart rate (HR), and arterial oxygen saturation (SpO_2_). The SOS button was the main subpart that signaled urgent help. The selector switch set the alarm at the lowest and highest HR and SpO_2_ in cases of unconsciousness. Although the general appearance of the device was designed the same as a general wristwatch or smart watch, it had a clear and large font displaying HR and SpO_2_. This enabled elderly people to see that this alert device was different from previous ones.

**Figure 2:**
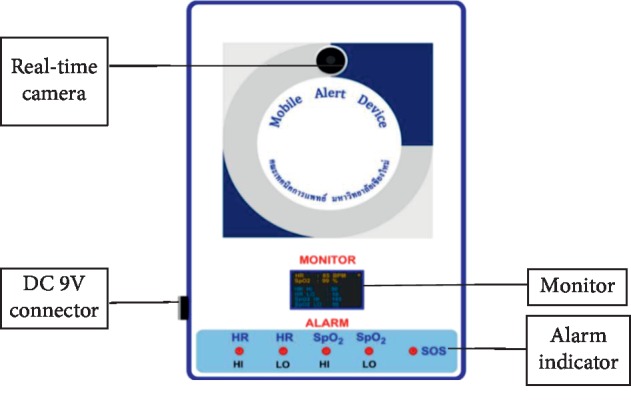
The signal receiving part of the emergency alert device.

The signal receiving part was a closed-circuit camera box connected to the Internet (as shown in Figure 2). There were four subparts, including a DC 9 V connector, alarm indicator, real-time camera, and monitor. This receiving part was set up at home and used domestic electricity with a DC 9 V Adaptor, which was connected to the DC 9 V connector subpart. The alarm indicator comprises five statuses such as SOS, HI-HR, LO-HR, HI-SpO_2_, and LO-SpO_2_. The real-time camera was able to provide two-way communication between the elderly or disabled people and their caregivers. This part provided comfort and safety until the helpers could arrive with help. Furthermore, the caregivers were able to locate dependents who might be unconscious. The monitor could show the data display in the same way as the signal sending part. Thus, the caregivers also were able to check the alert settings and monitor the HR and SpO_2_ of the elderly or disabled people. The general appearance of the receiving part looked like a general closed-circuit camera, but the novelty of this part was its HR and SpO_2_ display of set up alert scales and recent status of the sending part, which enabled the caregivers to double check a situation. In addition, it had HR and SpO_2_ alarm indicators that enabled the caregivers or helpers to know what caused an alarm.

#### 3.1.2. Functions and Alert System of the Device

**Figure 3:**
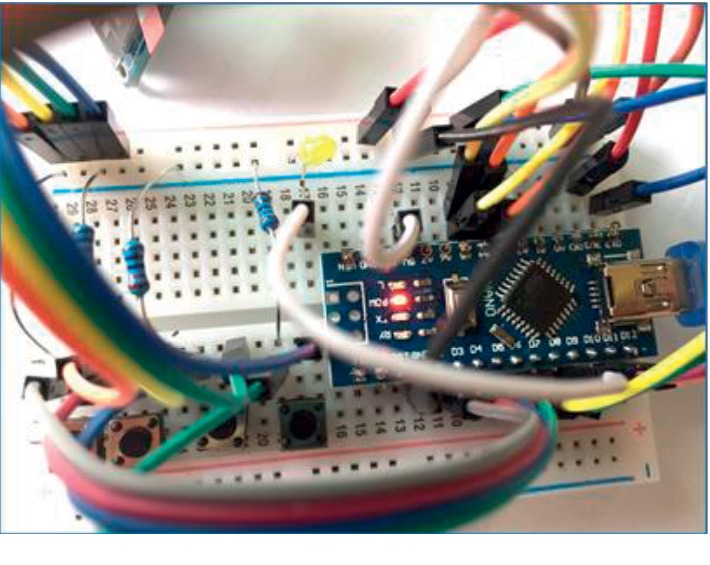
Assembly of the prototype electrical circuit.

**Figure 4:**
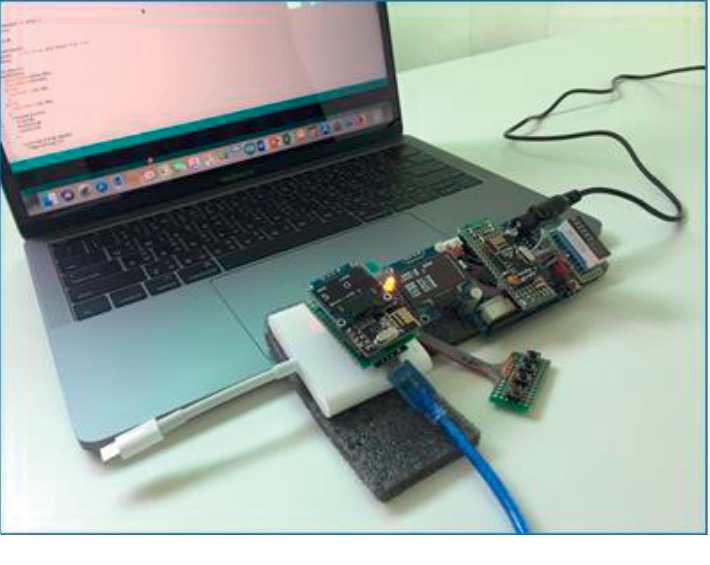
Uploading, testing, and adjusting the command program in the microcontroller.

After designing the general appearance and displays of the device, the mechanics of the sending part were operated, with their process generated in the same steps as those in general alert devices. However, the novelty of this device was that the receiving part not only received a signal from pressing an emergency button but also from a sensor. That is to say, besides a main processor and microcontroller, the HR and SpO_2_ sensors were built in the sending part of this device. The electrical circuits assembled in the prototype are shown in Figure 3. After the electrical circuits were assembled, a command prompt was set by C programming language (as shown in Figure 4). The command program was set in the main processor and microcontroller among the sending and receiving parts. Indeed, data from the SOS button showed that sensor readings of the user's abnormal HR or SpO2 proceeded within the main processor and microcontroller of the sending part and were transmitted via Bluetooth to the main processor and microcontroller of the receiving part.

**Figure 5:**
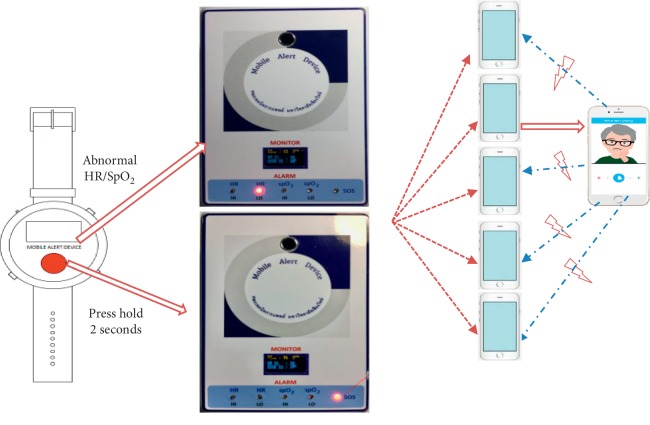
The alert system of the device.

There were two ways to make an emergency call such as conscious alert by pressing the SOS button and unconscious alert by HR and SpO_2_ monitoring. When elderly people or people with disabilities had an emergency accident at home, they were able to request help by pressing the SOS button. In order to prevent unintentional pressing, the SOS button was designed with a slightly depressed-curved surface that had to be pressed for two seconds. In addition, if there were serious problems with abnormal HR and SpO_2_, the SOS button worked automatically and sent a signal to the receiving part. In both methods for requesting help, conscious and unconscious alert, the sending part sent a signal to the receiving part. Then, the signal went through the Internet and Clever Dog Application immediately to five mobile phone numbers belonging to the caregivers. When a call was accepted, the remaining four phone calls stopped automatically. When a mobile phone accepted a call, sound and a picture determined whether the caller was requesting help consciously or they were unconscious. When pressing the SOS button, a flashing red light blinked SOS on the alarm indicator, and HR or SpO_2_ at HI or LO would light up if those readings were abnormal. The systematic functions of the alert device are shown in Figure 5. In addition, once the button for help had been pressed and the signal was accepted, the caller should wait for 30 seconds before pressing again. This system was designed to prevent overrepeated signals.

In terms of setting up the HR and SpO_2_ for automatic alert, the users could start by turning on the sending part. Then, the data display would be shown on the dial. Next, the selector switch was turned for a high- or low-alarm setting and then turned again to save the new settings.

### 3.2. Usability Testing of the Device

In the second phase of this study, the prototype device was tested by 12 specialists who worked in areas of medical and community services and assistive devices and 161 users comprising 146 elderly and disabled people and 15 caregivers in the community. The results of usability testing from the perspectives of the specialists and users are as follows.

#### 3.2.1. Usability Testing from the Perspectives of the Specialists

Data were collected from individual interviews on two aspects: general appearance and usability. In terms of general appearance, all five specialists reflected that the device had appropriate weight and materials, and the position and size of the SOS button was suitable. However, they commented that the size of the dial was too large for comfort and the letters displayed were too small to read clearly, especially for elderly people. They suggested that using symbols on the dial would be better in a limited area and easy to understand. Moreover, they recommended that the color of the letters or symbols be in contrast to the base of the dial, and in their opinion, the design of the wristband was old fashioned. Although this device was developed for elderly people and people with disabilities, fashion should be kept in mind. In terms of usability, all five specialists reflected that the device was easy and convenient to turn on or off with appropriate force to press the SOS button. However, they stated that people with hemiplegia or those with one hand might have limitations in using the device. They also recommended an elastic strap worn on one wrist. In addition, although the specialists agreed with the ease and convenience of connecting the device to the software application and charging the battery, they thought that it might be difficult for people with cognitive problems. However, this step could be performed by the caregivers, which would alleviate a serious problem. On sending a signal, the specialists gave their opinions on alternative ways of sending emergency signals such as voice recognition, short speech command, light touch, and so on. The perspectives of the specialists on the beneficial points of the device and their suggestions for improvement are summarized in Table 1.

#### 3.2.2. Usability Testing from the Perspectives of Users

The users who tested this device comprised 146 elderly people or people with disabilities and 15 caregivers or community health volunteers. Their perspectives on the device were collected by the rating scale usability checklist, which had five levels (5 = strongly agree, 4 = agree, 3 = neutral, 2 = disagree, and 1 = strongly disagree) comprising two aspects: general appearance and usability, as in the perspectives of the five specialists. The results showed that the users agreed with the general appearance and usability of the device. In terms of general appearance, the top three agreeable parts of the general aspect were the position and size of the SOS button, weight of the device and size of letters, and symbols on the dial, respectively. However, the lowest mean of the general appearance was physical such as size, dial, and type of wristband, to which the users showed a neutral level of agreement. In addition, the users agreed on the top three parts in the usability aspect, particularly the ease or convenience in connecting the device to a software application, the ease in pressing the SOS button, and the appropriate force needed to press the SOS button for sending a signal, respectively. The mean and standard deviation of each item in both sets of aspects (general appearance and usability) are shown in Table 2.

Although all of the users reflected the worthiness and usefulness of the device, they gave further interesting opinions. In terms of general appearance, they reflected on the points of weight, size, type of wristband, and color and brightness of the letters displayed. Indeed, they needed less weight and a smaller sized sending part with an elastic or a Velcro wristband. They also wanted the color of the letters to be in contrast to the base of the dial or the letters displayed to be brighter. Moreover, they gave an opinion about awareness of comfortable feeling when wearing the device: that it should be like wearing a general accessory and not look special or like weird medical equipment. In terms of usability, the users reflected that this device was useful for dependent people who want to stay safely at home and the caregivers who would have less stress and worry when being away from their dependents. However, they thought that the time it took to request help (30 seconds) was too long. Moreover, they thought it would be more practical and comfortable if a tiny camera was set up inside the sending part in order to send an emergency signal by lightly touching the screen.

## 4. Discussion and Conclusion

The development of emergency alert devices has continued with various types and functions. Most of the devices have been imported and sold in both medical equipment and online stores and designed for requesting help from nurse stations or caregivers at home [14]. These request signals are usually a short message, picture, or video sent via the Internet cloud server and linked to smart phones. This system is useful in reaching or accessing help for elderly people or people with disabilities in cities or those at a highly educated and socioeconomic level. However, some smaller communities might not be able to use the system so effectively. According to the World Health Organization (WHO) on healthy aging, every person in the world, including elderly people and people with disabilities, should have the opportunity to live a long and healthy life [15]. Such people in rural areas or suburban communities in Thailand have less opportunity to use hi-tech devices, including the emergency alert device, and most elderly people perform their daily activities alone. In addition, the percentage of performance in daily activities when alone was highest in the low active aging group [16].

The prototype of this device was designed with a familiar wristwatch appearance, which helped the users feel comfortable and not like a robot with a special device. This conceptual idea that elderly and disabled people are a sensitive group needs to be accepted by the general public. Acceptance and use of special devices are not easy for this group of people; thus, a device designed like a familiar object is more acceptable. Furthermore, a closed-circuit camera is currently not too expensive to set up in the home, and it generally links to personal mobile phones, which records any event that might occur when nobody is around to help. Therefore, it is possible and not difficult to connect an emergency alert device to a camera for sending requests for help to caregivers via a mobile phone and the Internet. However, most emergency technologies are used by users who are in a conscious state. Some devices are able to detect health behavior and predict risks, and others can detect falling and alert the nurse or caregiver, but there are currently fewer devices that can detect cases of unconsciousness in the home. The function of the emergency alert device in this study was designed to give the alarm for both conscious and unconscious cases. With this device, conscious users are able to press the SOS button, and abnormal HR and SpO_2_ can be detected when dependent users are unconscious. There were two points about the novelty of this device. First, it had the HR and SpO_2_ sensor as an important component that worked with the main processor and microcontroller in the sending part. Thus, although the users may be in an unconscious state or have abnormal HR and SpO_2_, the emergency alerts will work, while recent devices have set the system to receive emergency data from pressing a button or from a heavy falling detector. Second, the receiving part is not only a closed-circuit camera, but it also has an internal processor and microcontroller for connecting to and interpreting data from the sending part. Therefore, the receiving part can display the HR and SpO_2_ as shown on the sending part. Moreover, it can show the cause of the unconscious alert by a flashing red light on the receiving part so that the caregivers or helpers can give information to physicians or related health professionals.

The International Classification of Functioning, Disability, and Health (ICF) describes the products and technology as environmental factors that influence human functioning in people with and without disabilities [17]. However, not all technologies can be a facilitator for different people, as it depends on their differing needs, disabilities, daily activities, roles, and so on. Suitable technology is a facilitator that improves and encourages people to participate in meaningful activities [18–20]. Therefore, usability testing in this study was examined by specialists and users. Their perspectives were on the same points. The position and size of the SOS button and the force needed to press it was in strong agreement between the users and specialists. This was because the SOS button was put in the middle of the dial and big enough for elderly people to see it clearly. In addition, the button needed little force when being pressed, which was suitable for elderly and disabled people with limited hand muscle strength. These agreements were related to analysis of the needs and limitations of the users before designing the device and reflected that the appearance of AT was a point of awareness in the step of development [7]. In terms of suggestions for improvement, the specialists and users had the same perspectives on size of the dial, color and contrast of letters to the base of the dial, type of strap, and ways of sending signals. In this study, the size of the dial was as small as possible because it was related to the size of the electronic board inside it. In fact, a limitation of this study was the small size of the electronic board, which increased the amount of funding needed. Therefore, the general appearance of the device might have to be larger than a general wristwatch.

The caregivers gave an interesting perspective on the device as they said it relieved stress and helped them relax when needing to leave elderly and disabled people at home alone. This indicated that the emergency alert device is an AT that not only encourages dependent people to function but also increases the quality of life of the caregivers [5, 21, 22]. Although the device had points for improvement, all of the participants agreed with its development and the need to use it. This was because elderly people without disabilities had no legal right to have AT in Thailand, while most people with disabilities in the community had legal rights to receive it, but it mostly comprised services only and low-tech devices [8]. Therefore, specific hi-tech devices were lacking, and when the opportunity arose to try them, an interest and a need to use them in real situations were created.

There were limitations in this research in that it was a pilot study with a limited budget. The innovative output was a prototype product that was transpired from the idea of a trial device that might be developed for commercial use in the future. As a result, improved guidelines for developing an emergency alert device can be analyzed in the context of the users, such as physical limitation and conditions of the caregivers, systematic planning, continued training, and production costs [23, 24]. Furthermore, as the usability testing process was collected by various stakeholders and carried out in one community, generalization for other communities might be limited.

## Figures and Tables

**Table 1:** Summary of perspectives from the specialists on the prototype of the emergency alert device.

Usability	Advantages	Suggestions for improvement
General appearance aspect	(i) Light weight (ii) Made from suitable materials (iii) Appropriate position and size of the SOS button	(i) Decrease the size of the dial (ii) Decrease the thickness of the device (iii) Increase the letter display (iv) Use symbols (v) Use color of letters or symbols in contrast to the base of the dial (vi) Be aware of a fashionable design to enable a comfortable feeling when wearing outside the home

Usability aspect	(i) Easy and convenient to turn on or off (ii) Appropriate force used to press the SOS button	(i) Be aware of using one hand (ii) Be aware of the elderly and disabled people with cognitive problems setting up a software application, connecting to the Internet, and charging a battery (iii) Create alternative ways of sending emergency signals such as voice recognition, short speech command, and light touch

**Table 2:** Usability testing of the emergency alert device from the perspective of the users (*n* = 161).

Usability	$\bar{\text{X}}$ ± SD	Interpretation
*General appearances aspect*	4.11 ± 0.90	Agree
(1) Weight of the device	4.29 ± 0.76	Agree
(2) Physical appearance such as size, dial, and type of wristband	3.34 ± 0.71	Neutral
(3) Material of the device	4.16 **±** 0.95	Agree
(4) Position and size of the SOS button	4.48 **±** 0.84	Agree
(5) Size of letters and symbols on the dial	4.25 **±** 0.87	Agree
(6) Color and brightness of letters in contrast to the dial	4.14 ± 0.84	Agree

*Usability aspect*	4.37 ± 0.83	Agree
(1) Easy or convenient to wear	4.24 ± 0.78	Agree
(2) Easy or convenient to turn on or off	4.11 ± 0.85	Agree
(3) Easy or convenient to connect the device to a software application	4.86 ± 0.51	Strongly agree
(4) Easy to press the SOS button for sending a signal	4.41 ± 0.75	Agree
(5) Appropriate force to press the SOS button for sending a signal	4.33 ± 0.89	Agree
(6) Easy to charge the battery	4.27 ± 0.94	Agree

*Overall aspect*	**4.24** **±** **0.88**	Agree

## Data Availability

The data used to support the findings of this study are available from the corresponding author upon request.
